# On the evolutionary origin of aging

**DOI:** 10.1111/j.1474-9726.2007.00281.x

**Published:** 2007-04-01

**Authors:** Martin Ackermann, Lin Chao, Carl T Bergstrom, Michael Doebeli

**Affiliations:** 1 Institute of Integrative Biology, ETHZ 8092 Zürich, Switzerland; 2 Department of Biological Sciences, University of California San Diego, La Jolla, CA 92037, USA; 3 Department of Biology, University of Washington Seattle, WA 98195, USA; 4 Department of Zoology and Department of Mathematics, University of British Columbia Vancouver, BC, Canada V6T 1Z4

**Keywords:** aging, asymmetry, bacteria, damage, evolution, repair

## Abstract

It is generally believed that the first organisms did not age, and that aging thus evolved at some point in the history of life. When and why this transition occurred is a fundamental question in evolutionary biology. Recent reports of aging in bacteria suggest that aging predates the emergence of eukaryotes and originated in simple unicellular organisms. Here we use simple models to study why such organisms would evolve aging. These models show that the differentiation between an aging parent and a rejuvenated offspring readily evolves as a strategy to cope with damage that accumulates due to vital activities. We use measurements of the age-specific performance of individual bacteria to test the assumptions of the model, and find evidence that they are fulfilled. The mechanism that leads to aging is expected to operate in a wide range of organisms, suggesting that aging evolved early and repeatedly in the history of life. Aging might thus be a more fundamental aspect of cellular organisms than assumed so far.

## Introduction

Aging is an increase in intrinsic mortality and a decrease in reproductive rate with age (Rose, 1991). This deterioration is based on changes in tissues, cells and subcellular structures over time (Stadtman, 1992; Dice, 1993; Martin *et al*., 1996; Ashok & Ali, 1999; Partridge & Gems, 2002; Kirkwood, 2005b). If an aging organism reproduces, the structures that change over time are not equally distributed (Jazwinski, 1993; Lai *et al*., 2002; Ameisen, 2004; Kirkwood, 2005a). Rather, most or all of the aged structures segregate to one individual, called the ‘parent’. The other individual (the ‘offspring’) obtains structures that are newly synthesized and thereby resets its biological clock. In aging organisms reproduction is rejuvenating (Ackermann *et al*., 2003).

Such a distinction between an aging parent and a rejuvenated progeny probably did not exist in the first cellular organisms on earth. These organisms were unicellular and reproduced by dividing into two supposedly indistinguishable cells (Nystrom, 2003). In such organisms, there is no individual that persists across the cell division. This mode of reproduction precludes aging: if such organisms would deteriorate over successive generations, this deterioration would affect all individuals of the lineage simultaneously, and the lineage would disappear (Rose, 1991; Kirkwood & Austad, 2000). To prevent this, organisms without rejuvenating reproduction must avoid accumulation of cellular damage by repairing or renewing their structures to maintain their functioning. This does not mean that such organisms could not have substantial levels of genetic and cellular damage. It means, however, that such damage must remain approximately constant over successive generations, because any accumulation of damage would affect the whole lineage.

Aging, that is, accumulation of damage in individual organisms, could thus only evolve together with rejuvenating reproduction. Until recently, aging had only been described in eukaryotes (Mortimer & Johnston, 1959; Rose, 1991; Barker & Walmsley, 1999), and it was assumed that aging evolved after the origin of eukaryotes. However, two recent studies reported aging in bacteria (Ackermann *et al*., 2003; Stewart *et al*., 2005). Bacteria often do not distribute their subcellular structures equally upon division (Shapiro *et al*., 2002). Rather, many of the structures of the predivisional cell segregate to one progeny cell, while the other progeny cell synthesizes new structures. If the cell inheriting old structures is followed over many successive rounds of division, one finds that its division rate and growth rate decline (Ackermann *et al*., 2003; Stewart *et al*., 2005). This cell can thus be viewed as an aging mother that produces rejuvenated progeny. Individuality is not completely lost upon cell division. The progeny cell that inherits more old structures is more closely associated with the cell that underwent division than the other progeny cell. This reproductive asymmetry thus marks an important evolutionary step: it leads to the emergence of an individual that persists over successive generations, and this individual ages and produces rejuvenated progeny.

These experiments indicate that aging originated in very simple unicellular organisms. To understand this evolutionary transition, we thus have to understand two things: why such organisms would distribute aged, damaged structures unequally at reproduction (i.e. why there is rejuvenating reproduction), and why they would not repair such structures sufficiently to guarantee unlimited functioning. Here, we use simple models to investigate the conditions that would lead to the unequal distribution of damaged structures and thus to rejuvenating reproduction and aging. The basic assumption of our models is that the ancestral state of damage distribution among progeny cells is symmetric, which seems reasonable at least for early prokaryotic life (see above).

While these models are used to investigate the origin of aging in unicellular organisms, they are more general. They investigate evolutionary consequences of phenotypic damage. The defining aspect of phenotypic damage is that it is not necessarily copied upon reproduction. This contrasts with genetic damage, which cannot simply be reset during division. The evolutionary consequences of genetic damage form an active field of research. Our analysis of the evolutionary consequences of phenotypic damage complements these studies and provides a new perspective on the evolutionary origin of aging.

## Results and discussion

### Outline of the model

**Fig. 1:**
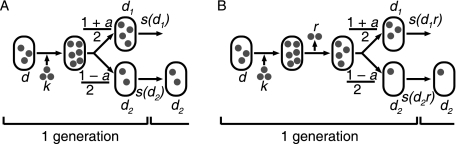
Outline of the model. (A) At the beginning of a generation, an individual contains some amount of damage *d*, represented by grey dots. It accumulates a further amount of damage, *k*, so that it contains a total amount of damage *d* + *k* before division. At division, the damage is divided among the progeny with a degree of asymmetry controlled by the parameter *a*. One of the progeny (top) obtains a fraction 0.5 * (1 + *a*) (*d* + *k*) (denoted as *d*_1_) of the damage, while the other obtains a fraction 0.5 * (1 – *a*) (*d* + *k*) (denoted as *d*_2_). After division, mortality is imposed. The first progeny survives with probability *s*(*d*_1_), the second with probability *s*(*d*_2_). In the example, the first progeny dies, and the second survives. All surviving progeny constitute the population at the beginning of the next generation. In the figure, damage is represented as discrete entities for simplicity; in the model, the amount of damage is a continuous quantity. (B) The model with repair. After accumulating an amount of damage *k*, the cell repairs part of the damage, *r*, so that the total amount of damage before division is *d* + *k* − *r*. The first progeny obtains a fraction 0.5 * (1 + *a*) (*d* + *k* − *r*) (denoted as *d*_1_) of the damage, the second progeny a fraction 0.5 * (1 – *a*) (*d* + *k* − *r*) (denoted as *d*_2_). The first progeny survives with probability *s*(*d*_1_*,r*), the second with probability *s*(*d*_2_*,r*).

We constructed a simple model describing a population of unicellular organisms (representing prokaryotes or simple eukaryotes) that reproduce by dividing into two progeny cells (Fig. 1A). At the start of a generation, each individual contains a certain amount of damage *d*, which was conferred to it by its parent, and which represents damage to subcellular structures, for example, oxidized proteins, or accumulated waste products. Before the next round of division occurs, each cell accumulates an additional amount of damage *k*, so that the total damage present in a cell before reproduction is *d* + *k*. At division, the phenotype *a* of a cell determines how the damage is distributed among its offspring: one of the progeny gets a fraction 0.5 (1 + *a*) (*d* + *k*) of the damage, while the other progeny gets a fraction 0.5 (1 − *a*) (*d* + *k*). Thus, *a* is a continuously varying phenotype between 0 and 1. A phenotype of *a* = 0 corresponds to symmetric damage distribution (both progeny get the same amount of damage), whereas *a* = 1 corresponds to fully asymmetric distribution (one progeny gets all the damage, and the other none). Both progeny inherit the phenotype *a* and go through the same cycle.

A population consisting of individuals of a given phenotype *a* will develop a stable ‘damage distribution’. A population where all individuals distribute damage symmetrically (*a* = 0) will converge to a state where all individuals will have damage *k* after division. The damage will be increased to 2*k* prior to the next division, which will then again result in two cells with damage *k* (Supplementary material). The amount of damage per cell reaches a stable value and does not continue to increase over time because the cellular damage investigated here is diluted with division; the purging action of natural selection is not needed to reach equilibrium.

In a population where all individuals have fully asymmetric damage distribution (*a* = 1), eventually all individuals will belong to one of the damage classes 0, *k*, 2*k*, 3*k*, etc. With asymmetric damage distribution, it is possible to identify an individual that persists over cell division. The life cycle of an individual starts as a cell that is produced damage-free, and that cell accumulates damage *k* over each successive round of division. Asymmetric damage distribution thus leads to an aging lineage with rejuvenating reproduction (Supplementary material).

### Asymmetric distribution as a strategy to cope with damage

Given a negative effect of damage on fitness, one can investigate the conditions that favor the evolution of asymmetric damage distribution and thus aging. We first consider the case where the damage *d* present in a cell emerging from division reduces the chance that the cell survives to the next division. Let *s*(*d*) denote the chance of survival as a function of the damage *d*. We initially assume that *s*(*d*) is linear: *s*(*d*) = (1 − *d*/*d*_0_) for some constant *d*_0_. A cell that distributes its damage symmetrically will produce two daughter cells with equal and intermediate amounts of damage, and hence with equal and intermediate survival probabilities. With asymmetric damage distribution, one of the progeny obtains less damage and experiences a higher survival probability, while the other progeny obtains more damage and thus suffers a lower survival probability. Because the relationship between damage and survival is linear, the increase in survival probability of the first progeny is identical to the decrease in survival probability of the second progeny. Therefore, the expected number of progeny surviving (the sum of the two individual survival probabilities) is unaffected by asymmetry. From this consideration, it might seem that the degree of asymmetry of damage distribution is a neutral trait.

**Fig. 2:**
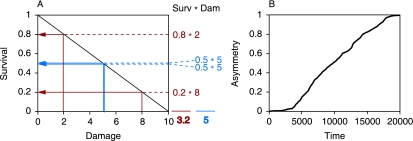
Evolution of asymmetric distribution of damage depends on the relationship between damage and survival. Asymmetric distribution of damage is favored if the relationship between the damage *d* inherited by a progeny and the chance *s*(*d*) that it survives to the next cell division is linear [0.5 *s*(*d*) = 1 − *d*/*d*_0_; *d*_0_ = 10]. A cell with damage 10 can distribute its damage symmetrically to produce two progeny with damage *d* = 5 and survival *s*(5) = 0.5 (blue arrows). Alternatively, it can divide asymmetrically, for example, producing a progeny with damage *d* = 2 and survival *s*(2) = 0.8, and a second progeny with damage *d* = 8 and survival *s*(8) = 0.2 (red arrows). The expected number of surviving progeny is one, independently of the level of asymmetry. However, asymmetric damage distribution increases the average quality of the surviving offspring. The expected sum of damage in the surviving progeny is 5 (0.5 * 5 + 0.5 * 5) with symmetry, but only 3.2 (0.8 * 2 + 0.2 * 8) with asymmetry (calculated as shown on the right side of the graph). (B) This advantage for asymmetric distribution of damage drives the evolution of asymmetry in a population with initially symmetric damage distribution. The graph shows results of the simulation model see Experimental procedures; population size *N* = 10 000, *d*_0_ = 10, damage accumulation *k* = 5, mutation rate µ*_a_* = 0.001, mutation size σ*_a_* = 0.01). This example shows a typical outcome where full asymmetry is fixed within 20 000 generations.

However, asymmetric damage distribution has another consequence that makes it inherently advantageous: with asymmetry, the progeny that is more likely to survive is also less damaged. Therefore, the expected quality of the offspring that survive to reproduction in the next generation is higher than with symmetric distribution (Fig. 2A). As a consequence, the expected number of descendents left after two generations is greater for individuals that distribute damage asymmetrically. This effect accumulates over the generations and allows lineages with asymmetric distribution of damage to grow faster than lineages with symmetric distribution. Therefore, higher values of the phenotype *a* should be selectively advantageous with a linear survival function *s*(*d*). By distributing the damage asymmetrically, selection is made to do something useful for the lineage (namely, disproportionately removing damage), instead of simply killing individuals all of whom are similar in the degree of damage.

In principle, given the function *s*(*d*) one can calculate the stable damage distribution, and hence the average fitness of each phenotype *a*. This turns out to be analytically intractable except in the special cases (Supplementary material), and we therefore use simple simulation models to confirm the inherent benefit to asymmetry with linear *s*(*d*). In these models, individuals accumulate and distribute damage as described above, which determines survival probabilities in each generation. The degree of asymmetry in the distribution of damage is a continuously varying trait. At division, progeny inherit the asymmetry phenotype from their parents subject to small mutations. The evolutionary dynamics generated by this model corresponds to a simple optimization: the winning phenotype is the one with the highest average fitness. This model shows that phenotypes with asymmetric distribution of damage always prevail over phenotypes that distribute damage symmetrically if the survival function *s*(*d*) is linear (Fig. 2B). For this case, it can also be shown analytically that the fully asymmetric type *a* = 1 has a higher growth rate than the symmetric type *a* = 0 (Supplementary material).

**Fig. 3:**
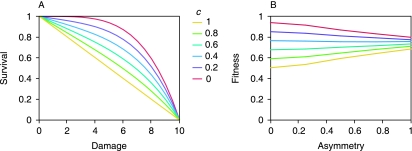
The advantage of asymmetry decreases for damage-survival curves that are concave down. (A) Six different damage-survival curves of the form 1 − *c* · *d*/*d*_0_ – (1 – *c*) (*d*/*d*_0_)^4^ are investigated; *c* is varied from 0 to 1 in increments of 0.2 (*d*_0_ = 10). (B) For each of the six curves from C, fitness is calculated as a function of the asymmetry of damage distribution (*k* = 5). For *c* = 0.4 fitness is largely independent of the level of asymmetry. To calculate the fitness of a phenotype with a given asymmetry under a defined damage-cost curve, we used the simulation model to determine the average fitness of monomorphic populations (see Experimental procedures).

Since linear survival functions *s*(*d*) favor asymmetric damage distribution, it is clear that nonlinear *s*(*d*) that are concave up (positive second derivative) also favor asymmetry, since for such survival functions asymmetry not only increases the expected quality of surviving progeny, but also the expected number of surviving progeny. Thus, symmetry can only be favored if *s*(*d*) is concave down (negative second derivative). In this case, asymmetric types have a lower expected number of surviving progeny than symmetric types. If this effect is strong enough, it is expected to outweigh the benefit of asymmetry. This is confirmed by our simulation model: symmetric damage distribution leads to a higher fitness than asymmetric distribution if the curvature of s(*d*) is sufficiently negative (Fig. 3A,B). Overall, our simulations thus indicate that asymmetric distribution of damage is favored over symmetry unless the survival function *s*(*d*) has substantial negative curvature.

Similar results hold when damage affects fertility rather than survival, i.e. the time until a cell emerging from division will divide again. In fact, in this case the advantage to asymmetric distribution of damage is even bigger, because there is an intrinsic advantage to variation in division times even in the absence of a difference in the quality of the offspring (Supplementary material).

These results show that there is an inherent benefit for asymmetric damage distribution that is independent of whether damage acts on survival or fertility. This benefit to asymmetry has two components. The first component is that asymmetric distribution of damage leads to variation in damage and hence in performance among progeny. The second component is an association in performance across generations. Individuals that carry little damage have high performance; their progeny will inherit little damage and will thus also have high performance. An association in survival probabilities between parents and progeny means that progeny with high survival are more likely to be produced (because their parents survive to reproduction). A very similar effect arises if damage acts on fertility, making this argument general and valid in a range of biological situations.

The model analyzed here can be extended to encompass further scenarios about how damage segregates upon cell division. For example, it is possible that asymmetric segregation becomes successively more difficult with increasing damage concentration. This could be the case if damage diffused into the supposedly damage-free progeny once its concentration exceeds a threshold. Also, it is possible that damage in the predivisional cell would impair the synthesis of new cellular components, so that both cells emerging from division would be harmed. If asymmetry is incomplete, heavily damaged cells could no longer produce damage-free progeny. This would manifest as a decrease in the condition of offspring born to damaged or old parents. Such an effect has been reported from diverse organisms ranging from bacteria (Stewart *et al*., 2005) to humans (Gavrilov & Gavrilova, 2000).

### Asymmetric damage distribution as an alternative to repair

Up to this point we have assumed that damage that arises in a cell is passed on to its progeny rather than being repaired. However, many types of cellular damage can be repaired (Kirkwood, 1981; Kirkwood & Rose, 1991; Clarke, 2003). Waste products can be degraded or excreted. Deleterious changes in subcellular structures can be repaired (Schroder *et al*., 1993) or the structures replaced (Hershko, 1982). Will asymmetry still evolve if damage can be repaired?

To answer this question, we introduced repair as a phenotypic trait in the simulation model (Fig. 1B). Specifically, we assumed that if the repair phenotype of an individual is *r*, then the damage accumulated between two rounds of division is *k* – *r* (rather than *k*, as was the case without repair). It is likely that repair comes at a cost, resulting either from the manipulation of waste products or from the replacement or repair of subcellular structures. We therefore assumed that the repair phenotype *r* carried a cost in survival probability, so that the probability that a cell with damage *d* and repair *r* survives, *s*(*d*,*r*), is the probability that it survives damage-induced costs times the probability that it survived repair-based costs. Repair has two opposing effects on survival. On the one hand, repair is costly and thus leads to a direct decrease in survival. On the other, repair decreases damage and thus leads to increased survival. For simplicity, we assume that increasing investment in repair has linear costs in terms of survival: *s*(*d,r*) = *s*(*d*) * (1 − *r*/*r*_0_), where *r*_0_ is a constant, and s(*d*) describes how survival declines with increasing damage. The results reported below do not depend qualitatively on the assumption of linearity.

**Fig. 4:**
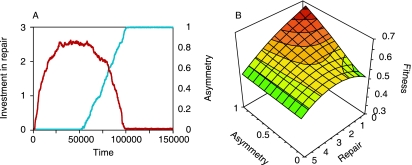
Interactions between asymmetric distribution of damage and repair. (A) Results of the simulation model when the level of asymmetry of damage distribution and the investment in repair were evolving phenotypic traits (population size *N* = 2000, *d*_0_ = 10, *r*_0_ = 10, *k* = 5, µ_*a*_ = 0.001, µ_*r*_ = 0.001, σ_*a*_ = 0.01, σ_*r*_ = 0.05). For the first 50 000 generations, asymmetry was kept at zero, and the investment in repair evolved to an equilibrium value. After generation 50 000, asymmetry was free to evolve and increased to a value of one, while the investment in repair decreases. (B) The fitness landscape for the situation described under A. The evolutionary interaction between asymmetry and repair is represented in the shape of the fitness landscape. With increasing asymmetry, the strength of selection for reduced repair increases (the slope in direction of reduced repair is steeper for larger asymmetry). On the other hand, with decreasing repair the strength of selection for more asymmetry increases. There is one global maximum at minimal repair and maximal asymmetry.

If we first assume that the damage distribution is constrained to be symmetrical (all individuals have phenotype *a* = 0), then the repair phenotype evolves to an intermediate value that maximizes the survival probability *s*(*d*,*r*). This value can be calculated analytically (Supplementary material). With the evolutionarily stable level of repair and symmetric damage distribution, the arguments presented previously for the inherent advantage of asymmetry again apply, and we thus expect an evolutionary change towards asymmetry. This is confirmed by the simulation model, in which asymmetric distribution of damage evolves in populations with initially symmetric distribution that have equilibrium levels of repair (Fig. 4A).

Interestingly, once asymmetry has evolved it becomes advantageous to decrease investment into repair (Fig. 4A,B). With asymmetric distribution of damage, substantial repair is no longer necessary, because cell division always leads to one cell with little or no damage. This generates a positive evolutionary feedback: increasing asymmetry leads to decreased repair, which in turn increases selection for more asymmetry (Fig. 4A,B). Very similar evolutionary dynamics are observed when damage affects fertility rather than survival (results not shown). Thus, distributing damage asymmetrically is an alternative to repairing it. Which of these two mechanisms will actually be used by organisms to control damage will depend on how costly these two processes are and how easily they evolve. For example, if direct costs for asymmetry are incorporated in the model, we find that a new (local) fitness optimum with symmetric damage distribution emerges (Supplementary material). However, as long as the costs for asymmetry are not very large, highest fitness, i.e. the global fitness maximum, is still achieved with asymmetry.

It has been suggested before that asymmetric distribution of damage could be an alternative to repair (Kirkwood, 1981; Stephens, 2005; Stewart *et al*., 2005). Our explicit model supports this hypothesis, and helps understand the causes for the advantage of asymmetry as well as the conditions under which this advantage emerges. Two other theoretical studies recently also investigated the conditions under which asymmetric distribution of cellular damage would be favored over symmetric distribution. The first study by Watve *et al*. (2006) assumed that damage accumulates in a number of cellular components that interact to determine cell functioning. In that model, damage decreases the efficiency of the cellular components. Their study reports advantages for asymmetry under more restrictive conditions than observed in our model. The discrepancy is a consequence of a number of differences between the two models. In the model of Watve and colleagues, mortality only affects asymmetrically dividing cells that contain components of the most damaged class. Asymmetrically dividing cells with components of intermediate damage classes and symmetrically dividing cells are free of mortality. Also, symmetrically dividing cells are capable of repairing damaged components, while asymmetrically dividing cells are unable to repair. Under those assumptions, asymmetry increases the growth rate if the efficiency of the cellular components decreases quickly with damage, and if cell productivity is determined by the most damaged component. This advantage vanishes if cellular components act independently to determine cell productivity, or if the efficiency of the cellular components decreases only slowly with damage.

The second study, by Evans & Steinsaltz (2006), also models the accumulation of damage that affects survival probability, and is thus closer to the scenario investigated here. In their model, intermediate levels of asymmetry are optimal, but large levels of asymmetry are disadvantageous. The main difference with our model is that in their model damage is not divided upon cell division. Rather, with completely symmetric division, each daughter cell receives an amount of damage that is equal to the damage in the predivisional cell. As a consequence, the amount of damage continuously increases, and cells with low amounts of damage can only be regenerated if damage is distributed unequally upon division or if it is repaired. The model starts off with a population that already exhibits random differences in the amount of damage between two cells emerging from division, and studies how selection acts on modifiers that increase the degree of the asymmetry.

These models complement one another to form a new theoretical foundation for understanding the evolutionary origin of aging in simple life forms. They cover a range of scenarios of how damage hampers functioning, and of how damage is passed on to the progeny upon division. The resulting theoretical insights will hopefully spur experimental research on the relationship between asymmetric cell division and aging. Such empirical studies might then reveal which model is best suited to describe the biology of a given organism.

### Biological considerations and experimental data

The main result of our theoretical analysis is that asymmetric damage distribution has an intrinsic advantage that manifests itself under a large range of conditions independently of how damage affects fitness components of the organisms. This indicates that aging might easily evolve in initially non-aging organisms under a wide range of conditions. The biological relevance of these findings hinges on the question of whether the conditions favoring asymmetric damage distribution are frequently met in real organisms, and if they are, whether asymmetric damage distribution can evolve. We address these two aspects in turn.

Our analysis shows that the advantage for asymmetric damage distribution depends on the form of the relationship between damage and fitness components; asymmetry is favored unless the rate at which fitness decreases with increasing damage is strongly accelerating with increasing damage. It is difficult to make a priori predictions about whether relationships found in real organisms would benefit asymmetric damage distribution, so empirical data are needed. As a first step in this direction, we used experimental data from bacteria to investigate how fitness components of individual bacterial cells decline with increasing physical age of the cells, which we used as a measure for the amount of cellular damage.

We employed an experimental system that was previously used to study aging in bacteria (Ackermann *et al*., 2003): by direct microscopic observation we can follow individual cells of the bacterium *Caulobacter crescentus* over a large number of consecutive divisions, and we can determine how the reproductive output of individual cells changes with age. Every cell division in *C. crescentus* is asymmetric. A sessile stalked cell divides to give rise to a motile swarmer cell. After cell division, the stalked cell immediately initiates a new round of division, while the swarmer cell differentiates into a stalked cell before dividing. Stalked cells of *C. crescentus* age manifested as a decline in the rate of cell division with increasing age (Ackermann *et al*., 2003). Assuming that this decline is a consequence of the accumulation of damage in the stalked cell, we used it as a proxy for estimating how fitness decreased with increasing damage.

**Fig. 5:**
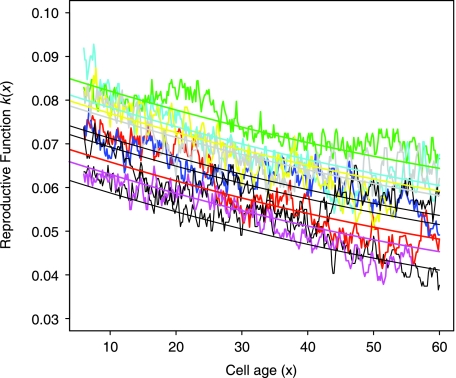
Decrease in fitness components with increasing age in cells of the bacterium *Caulobacter crescentus*. Reproductive function (moving average, window size 5 h) as a function of the cell age. Depicted are data from nine independent experiments where cohorts of 30 cells of *C. crescentus* were followed to an age of 60 h (corresponding to about 25 cell divisions), with the quadratic best fit. Both the quadratic and the linear term are significant (*P <* 0.001).

We investigated how the reproductive output in a cohort of stalked cells changed with physical age. Our measure for the reproductive output was the number of progeny produced per member of the cohort per unit time. This measure corresponds to the product of the probability of survival to a given age, multiplied by the fecundity at that age (see Experimental procedures). Using nine independent experiments where 30 stalked cells were followed for 60 h (corresponding to about 30 cell divisions), we determined the relationship between reproductive output and age (Fig. 5). Assuming that damage accumulated at a constant rate with age in the stalked cell, we transformed this information into a function-relating damage to fitness components. The form of this function is close to linear but slightly concave up (Fig. 5); based on our results, one would thus conclude that an asymmetric damage distribution would be favored.

Implementing the empirically estimated fitness function in our theoretical model, we found that this is indeed the case: the model predicts that for the *C. crescentus* system, asymmetric distribution of damage leads to a higher growth rate compared to symmetric damage distribution. The effect is statistically significant (Mann–Whitney U-test, *P* < 0.001) but very small: asymmetric damage distribution leads to a fitness increase of 0.06% compared to symmetric damage distribution. The likely reason for the small difference in fitness between asymmetric and symmetric damage distribution is that this bacterium ages very slowly (Ackermann *et al*., 2003) and thus accumulates damage at a slow rate, so that a change in the distribution of this damage does not have a large effect on fitness.

This analysis is based on the assumptions that the decline in reproductive output in *Caulobacter* stalked cells is a consequence of damage accumulating in the cell, and that the physical age (from differentiation) of a stalked cell is a good proxy for the amount of cellular damage. While these assumptions await further validation, our experimental observations of *C. crescentus* provide first evidence that the relationship between damage and fitness in this bacterium is of a form that favors asymmetric damage distribution and thus aging, which is in line with the observation that this bacterium indeed ages (Ackermann *et al*., 2003).

The next step will be to conduct experiments with single cells where the amount of intracellular damage can be quantified, and its consequences on reproduction and survival assessed. The experimental system that is currently most advanced is the budding yeast *Saccharomyces cerevisiae*, where proteins that are damaged in the course of aging can be identified (Reverter-Branchat *et al*., 2004), the extent of oxidation and carbonylation can be quantified (Cabiscol *et al*., 2000; Aguilaniu *et al*., 2003) and the consequences of a loss of asymmetric inheritance of damage can be studied (Lai *et al*., 2002; Aguilaniu *et al*., 2003). Similar techniques are also available for bacteria, where damaged proteins (Dukan & Nystrom, 1998) and protein aggregates (Mogk *et al*., 2003) can be identified and quantified. Such data gained from the observation of individual cells could be used to test the predictions that emerge from the model. One prediction is that there is an association between the damage acquired by subcellular structures over several cell divisions, and the chance that these structures are asymmetrically inherited. For example, proteins that are damaged in the course of aging (as measured in *S. cerevisiae*, Reverter-Branchat *et al*., 2004) are expected to be distributed asymmetrically. Proteins that are asymmetrically distributed can be identified by global analysis of protein localization (Huh *et al*., 2003).

The second biologically relevant question is whether mutations that facilitate asymmetric damage distribution actually do occur in simple unicellular organisms. Asymmetric distribution results if damage is localized to specific positions in the cell rather than diffusing freely. One way of achieving this is to localize damage to the cell pole (Ackermann *et al*., 2003; Stewart *et al*., 2005). Many unicellular organisms have two defined cell poles to which they localize subcellular structures (Shapiro *et al*., 2002; Janakiraman & Goldberg, 2004). Recent experimental work uncovered core mechanisms that have been adapted in a diverse range of cells for the generation of cell polarity and thus asymmetry (Nelson, 2003). One structure facilitating the localization and asymmetric distribution of molecules is the cytoskeleton, which has recently also been described in bacteria (Errington, 2003). Overall, unicellular organisms have a substantial degree of subcellular organization and polarity. As a consequence, it seems likely that mutations leading to asymmetric distribution of waste or damaged structures can readily occur.

Asymmetric partitioning of waste or damaged structures does not necessarily require that these entities localize to specific positions in the cell. An alternative mechanism is that damaged structures or waste aggregate to one cluster, and that this aggregate is passed to one of the two progeny upon division. A recent study demonstrated that asymmetric inheritance of damaged proteins in cells of higher eukaryotes is based on this mechanism (Rujano *et al*., 2006). This form of asymmetry is concordant with the model analyzed here, and could thus evolve for the reasons outlined above. It does, however, lead to a conceptual and experimental difficulty. We defined the ‘parent’ as the cell that inherits more of the old or damaged structures. In the scenario discussed here, the segregation of the aggregate would determine which of the two cells emerging from division should be considered as the ‘parent’. This classification is problematic if different subcellular structures segregate independently from one another. For example, if some damaged or old structures localize to the cell poles, and other damaged structures form an aggregate that segregates independently from the poles, then an unequivocal distinction between ‘parent’ and ‘offspring’ is not possible. It will be interesting to test experimentally whether such a situation is frequent in biological systems, or whether the majority of old or damaged parts tends to segregate together.

### An addition to the classic theory

The theory presented here complements the classic theory for the evolution of aging (Medawar, 1952; Williams, 1957; Hamilton, 1966).While these classic theories of aging are based on the assumption of an existing separation between a parent and a rejuvenated offspring (for example, mediated by the germ-soma separation), one can interpret our results as explaining the origin of such a separation. This not only forms the basis for the mechanisms described by the classic theory, but goes one step further in explaining the origin of aging: in our model, the asymmetric distribution and incomplete repair of damaged structures already leads to aging. Asymmetric damage distribution leads to the emergence of a recognizable parent whose identity is not lost upon division, and that accumulates damage over consecutive rounds of division and thus experiences a decline in condition with increasing age. A senescent decline in performance late in life can thus evolve in this model without the need to explicitly invoke mutations with age-specific deleterious effects, as in the classic theory. Rather, decreases in age-specific rates of survival and fecundity are a consequence of the asymmetric inheritance of damage.

While our analysis was presented in terms of the distribution of subcellular damage in unicellular organisms, the main result is general: there is an inherent advantage to concentrating damaged structures and waste into one individual at reproduction, and letting the other emerge rejuvenated. Our model makes no specific assumptions that restrict it to unicellular organisms or to specific kinds of damage to subcellular structures. The conclusions can thus be extended to other types of organisms. In multicellular organisms, the parts that are distributed at reproduction are not subcellular structures, but rather whole cells, tissues and organs. One would thus predict an inherent advantage to segregating damaged cells and tissues to a parent and building a progeny from scratch. This is already realized in multicellular organisms with a clear separation of soma and germline. There, the damaged soma as a whole segregates to the parent, while the progeny builds a new soma out of the parent's germline from which it emerges.

## Conclusions

Our study suggests that natural selection can readily favor the evolution of a distinction between an aging parent and a rejuvenated offspring. The benefit of distributing damage asymmetrically is expected to be manifest under a wide range of conditions, irrespective of the type of damage and details of how damage affects the organisms functioning. Internal structures of bacteria and unicellular eukaryotes facilitate the localization of damage, which is a precondition for asymmetric damage distribution. The likelihood of asymmetry is increased by the fact that there are many different types of cellular damage. Even if for a majority of these types asymmetric distribution is not possible or not favored, there are presumably at least some types of damage for which asymmetric inheritance evolves, and this would be enough to initiate aging. Furthermore, the possibility of repairing damage does not prevent the evolution of asymmetric damage distribution, which is favored even if a part of the damage is repaired. These factors together might have driven an evolutionary transition in many organisms toward a state where one of the individuals emerging from reproduction receives all the phenotypic problems associated with old age, while the other emerges rejuvenated and with the full life potential. Our results raise the question of whether it is possible at all for simple unicellular organisms, be they prokaryotic or eukaryotic, to avoid the evolutionary emergence of aging.

## Experimental procedures

### Individual-based simulation model

We used individual-based simulation models to investigate the evolutionary dynamics of asymmetry of damage distribution and investment in repair, and also to determine how the fitness of a phenotype depended on these two traits. We modelled populations with a carrying capacity of *N* = 1000 individuals. Each individual was characterized by the two phenotypic traits asymmetry a and investment in repair *r*. In models without repair this trait was set to *r =* 0 for all individuals. Acquisition and distribution of damage was implemented according to the general model setup described above. We developed one model for damage acting on survival and one for damage acting on division time. In the first model, generations were synchronized. In each generation, individual survival probabilities were normalized so that the expected population size after division (i.e. at the start of the next generation) was equal to the carrying capacity. The normalization of survival probabilities effectively imposes a density-dependent mortality that affects all phenotypes equally. In particular, there is no frequency-dependence in this model, and the winning phenotype is thus simply the phenotype with the highest average fitness calculated from its stable damage distribution. For the second model, individuals were not synchronized, and population size was kept constant by randomly removing an individual from the population each time a division occurred. Importantly, the assumption of density-dependent population regulation does not influence how selection acts on asymmetry. We assumed density-dependent mortality that affects all individuals equally and thus leaves the age-distribution (or the damage-distribution, respectively) unaltered. Under this type of density-dependence, selection acts towards maximizing the population growth rate *r* (Mylius & Diekmann, 1995). The evolutionary outcome is thus the same as if we would assume periods of exponential growth alternating with episodes of high external mortality. Our conclusions are thus independent of any assumptions about the population dynamics, which makes them more robust.

When we used the individual-based model to investigate the evolutionary dynamics of asymmetry and repair, we started with monomorphic populations with zero asymmetry and zero investment into repair. At each cell division, these two traits mutated with probabilities µ_*a*_ and µ_*r*_, respectively. Mutations were drawn from normal distributions with mean zero and standard deviations σ_*a*_ and σ_*r*_, respectively. The model was then run for many generations and the outcome (i.e. the phenotypes present) recorded.

When we used the individual-based model to determine the fitness of phenotypes with asymmetry *a* and repair *r*, we initialized monomorphic populations of phenotypes with these particular trait values and let them reach stable damage-distribution. The fitness of the phenotype is then simply the growth rate of the population at equilibrium.

### Direct observation of individual bacterial cells

We used a method described in (Ackermann *et al*., 2003) to determine the reproductive output of individual stalked cells of the bacterium *C. crescentus* by means of direct observation under the microscope. We analyzed data from nine independent experiments with the wild-type strain UJ590 (Ackermann *et al*., 2003), each experiment initiated with a cohort of about 30 young stalked cells. At intervals of 10 min, we monitored the number of progeny produced per individual of the cohort. The number measured at a given age *x* corresponds to the product of the probability of surviving to that age, *l*(*x*), and the rate of reproduction at that age, *m*(*x*). This product is referred to as reproductive function *k*(*x*) (Charlesworth, 1994), which determines the fitness of a strain (the intrinsic growth rate). In this experiment, it is not possible to determine whether cells that do not divide are dead or rather alive but not reproducing. As a consequence, it is not possible to separate the reproductive function *k*(*x*) into its two components, survival *l*(*x*) and reproduction *m*(*x*). However, how this separation is done is inconsequential for the estimate of fitness of the strain. To reconcile these data with our models, we thus made the following two assumptions: The decline in the reproductive function *k*(*x*) with age observed in stalked cells is a consequence of damage that accumulates at a constant rate. Importantly, assuming a constant rate of damage accumulation in the stalked cell implicitly assumes that the damage that leads to the observed deterioration in condition with increasing age is distributed with full asymmetry. There is experimental evidence that this is the case: when following progeny produced by stalked cells in the flow chamber, we observed that progeny born to young mothers and progeny born to old mothers do not differ in performance, indicating that the progeny do not receive damage from their mothers (these measurements were done with a strain that showed increased attachment, and this strain also shows a relationship between age and the reproductive function that is concave up; details not shown). The damage in a cell determines its rate of reproduction, and has no effect on mortality. Technically, this corresponds to assuming that changes in *k*(*x*) with age *x* are solely a consequence of a change in *m*(*x*), and that *l*(*x*) was equal to 1 for all ages *x*. As stated above, this assumption does not affect the estimate of a genotype's fitness.

To extract the relationship between damage and fitness from the experimental data, we used procedure nls in the R statistical software (R Development Core Team, 2005) to determine a quadratic function *k*(*x*) with the best fit to the observed reproductive functions from the nine experiments. We fitted a common linear and quadratic term and individual intercepts for the nine experiments; the arithmetic mean of the nine intercepts was used as the intercept of *k*(*x*). *k*(*x*) was interpreted as the relationship between damage and the rate of progeny production. This allowed us to determine how selection acted on asymmetric damage distribution in a population subject to this function. We used the computer models to determine the growth rates of populations with asymmetric damage distribution and populations with symmetric damage distribution. Growth rates were determined by measuring growth over 20 generations in populations of 10 000 individuals. We performed 100 runs each for asymmetric and symmetric damage distribution.

For further details on the analytical model, see the Supplementary material.

## Supplementary material

The following supplementary material is available for this article:

Fig. S1 Fitness as a function of repair and asymmetry, with costs for asymmetry. (A) The fitness landscape if asymmetry has intermediate costs (*a*_0_ = 12). A local optimum with zero asymmetry and intermediate repair emerges. The global optimum remains at full asymmetry and zero repair. (B) With large costs for asymmetry (*a*_0_ = 5) the global optimum moves to zero asymmetry and intermediate repair; the fully asymmetric state is still a local optimum. To determine the fitness landscape, we used the simulation model to determine the long-term growth rate of a population monomorphic for different phenotype combinations of asymmetry and repair (see Experimental procedures).

Appendix S1 Supplementary model description.

This material is available as part of the online article from: http://www.blackwell-synergy.com/doi/abs/10.1111/j.1474-9726.2007.00281.x (This link will take you to the article abstract).
