# Supplementary material for: On the evolutionary origin of aging
Source: Aging Cell. 2007 Apr 1;6(2):235–44. doi: 10.1111/j.1474-9726.2007.00281.x (PMC2049046; doi:10.1111/j.1474-9726.2007.00281.x)
Supplement: Appendix S1 — Supplementary model description. [file ace0006-0235-as1.doc]

**Supplementary Material**

*1. General model setup*

We modelled unicellular individuals that reproduce by dividing into two progeny. Between two consecutive divisions, each cell acquires damage *k*, which is added to the damage *d* that the cell inherited during the last division. In the cases where repair was included in the analysis, the damage of a cell with investment *r* in repair is decreased by an amount *r* prior to division, resulting in damage *d* + *k* *-* *r*. Repair had costs in terms of survival, so that the survival probability *s*(*d*,*r*) of a cell with damage d and investment into repair r is equal to $s(d)\cdot(1-r/r_{0})$, where *r*_0_ is a constant, and *s*(*d*) describes how survival declines with increasing damage. When costs for asymmetry were assumed, survival probability s(d,r,a) of a cell with damage d, repair r and asymmetry a was assumed to be$s(d)\cdot(1-r/r_{0})\cdot(1-a/a_{0})$, where *a_0_* is a constant. None of the results reported depend qualitatively on the linearity assumptions for costs of repair and asymmetry.

*2. Survival depends on damage and investment into repair: stable damage distribution and optimal investment into repair.*

If damage is distributed symmetrically and not repaired, eventually all individuals have damage *k* at the beginning of each generation (i.e., the stable age distribution consists of a single damage type). This follows from the dynamics of damage at time t, d(t), which are as follows: d(t+1)=(d(t)+k)/2. At steady state, d(t+1)=d(t)=d*, so we have d*=d*/2 + k/2, i.e. d*=k. Thus, for symmetric damage distribution at reproduction, the stable damage distribution consists of a single damage class, and the average fitness of this symmetric type is then given by *s*(*k*).

Analogously, if all individuals repair an amount r of their damage, the stable damage distribution consists of all individuals having damage (*k* - *r*) at the start of their life cycle. The average fitness of this type is *s(k-r,r)*. The optimal level of repair is the value $\hat{r}$ that maximizes the survival function *s*(*k-r*, *r*). For example, if *s*(*d*) is linear, $s(d)=(1-d/d_{0})$, and s(d, r) = s(d) (1–r/r_0_) , then $\hat{r}=(k+r_{0}-d_{0})/2$. With symmetric damage distribution and repair, the repair phenotype always evolves to $\hat{r}$.

It might be surprising that the damage per individual reaches a stable equilibrium rather than continuing to increase over time. The reason for this is that the phenotypic damage accumulating over time is diluted with each division. The stable damage distribution is thus not a consequence of selection eliminating strongly damaged cells, but rather of this dilution process. This differs in an important way from the dynamics of accumulating damage to the informational content of the cell, such as mutational damage to the DNA. Mutational damage is not diluted at division; rather, it is copied prior to division, so that (in the absence of sex) both progeny end up with the full complement of mutational damage that was present in the parent. Mutational damage therefore requires the purging action of natural selection in order to reach a steady state.

*3. Survival depends on damage: advantage for asymmetry.*

When *s*(*d*) is linear, $s(d)=(1-d/d_{0})$, one can show analytically that the completely asymmetric phenotype *a* = 1 has a higher average fitness than the symmetric type *a* = 0, as follows. As shown above, with symmetric damage distribution the average fitness is given by *s*(*k*).

On the other hand, with full asymmetry, the two progeny of an individual starting out with damage *k* have damage (*d* + *k*) and 0, respectively, at the beginning of their life cycle. The progeny of these progeny then belong to the damage classes 0, *k*, *d* + 2*k*. Thus, after *n* generations, all descendents of the individual that started out with damage *d* belong to one of the damage classes 0, *k*, …, (*n* - 1) *k*, *d* + *nk*. As *n* gets large, the contribution of the class *d* + *nk* obviously becomes negligible, and hence the stable damage distribution of the fully asymmetric type contains exactly the age classes 0, *k*, 2*k*, …. The frequency with which these classes occur at equilibrium depends of course on the survival probabilities *s*(*nk*), *n* = 0, 1, 2,…, and can, in general, not be computed analytically.

However, we can make the following analytical argument to show that fully asymmetric types have higher average fitness than symmetric types when *s*(*d*) is linear. Suppose for a moment that a phenotype distributes damage fully asymmetrically, but that asymmetry does not affect the quality of the offspring, so that each of the two offspring of any individual has and equal chance of survival. Then it is again easy to convince oneself that at stable age distribution, damage class 0 occurs with frequency 1/2, damage class *k* occurs with frequency 1/4, class 2*k* with frequency 1/8, and in general damage class *nk* occurs with frequency $(1/2)^{n+1}$. Let us call this the neutral damage distribution. The average amount of damage in the population is the sum over all damage classes, weighed by the frequency of each class: ∑ nk*(1/2)^n+1^, which is equal to k*∑ n*(1/2)^n+1^. The sum ∑ n*(1/2)^n+1^ evaluates to 1, and the average amount of damage in the population is thus *k*. Because the function relating damage to fitness is linear, the average fitness of the “fictitious” neutral distribution is equal to the fitness of an individual with the average amount of damage, *k*. The average fitness is therefore *s(k),* which is equal to the average fitness of the symmetric type calculated above. (Note: strictly speaking, this calculation would require that all damage classes have positive survival, which is not true, because *s*(*d*) is linearly decreasing, but if the slope -1/*d*_0_ is small, the following argument is still approximately true.)

The point is now that when asymmetric damage distribution does have an effect on quality of offspring, as in our general model setup, then the stable damage distribution for a fully asymmetric type will obviously be shifted to lower damage classes compared to the neutral distribution, because offspring that are more likely to survive will also have lower damage. Therefore, the average fitness for the fully asymmetric type will be higher than for the neutral distribution, and hence higher than for the symmetric case. This proves that asymmetric types have a higher average fitness than symmetric types when *s*(*d*) is linear and asymmetry does not have costs.

*4. Damage affects fertility: advantage for asymmetry*

An alternative to assuming that damage affects survival to reproduction consists of assuming that damage *d* that is present in a cell emerging from division affects the time *t*(*d*) until the cell divides again. For the analysis of this case it is important to note that variation in division times of offspring confer an intrinsic advantage even if there is no association between division time and quality of offspring. To see this, let us consider a type that produces two progeny, one with a division time *t*_0_ + *t* and one with a division time *t*_0_ *-* *t*. Let us further assume that each of these two offspring again produce two progeny with those division times (thus, time to division of the parent does not affect the time to division of the progeny). It is easy to see that these assumptions are equivalent to assuming an organism with a life history in which the first division takes a time *t*_0_ + *t* (which could be considered age at maturity), and all subsequent divisions take a time *t*_0_ *-* *t*. Using the Euler-Lotka equation (Charlesworth, 1994), one can give an analytical expression for the intrinsic growth rate of such a life history. Based on this expression, it can be shown that the growth rate is a monotonically increasing function of the parameter *t*: the bigger the variation in time to division among the offspring, the higher the growth rate.

Thus, there is an intrinsic advantage to introducing variation in division times among the two offspring. If damage affects division time linearly, $t(d)=d_{1}\cdot(1+d/d_{2})$ (which means precisely that the offspring have division time *t*_0_ + *t* and *t*_0_ *-* *t* for some *t*_0_ and *t*, where symmetric damage distribution would lead to division time *t*_0_ ), then asymmetric damage distribution should be favoured for this reason alone. However, it should be additionally favoured because of the association between short division times and quality: the offspring that have short division times have less damage, and therefore themselves produce offspring with short division times.

To see that this additional affect is sufficient to favour an asymmetric damage distribution, one can control for the intrinsic advantage to asymmetric division times by choosing different damage function *t*(*d*). For example, one can show (again using the Euler-Lotka equation) that with $t(d)=c_{1}\cdot\exp[c_{2}\cdot d]$(where *c*_1_ and *c*_2_ are constants), the intrinsic advantage to asymmetric division times is lost (i.e., if there is no association between division times of parent and offspring, then types whose offspring have equal division times *t*(*d*) for some *d* have a higher growth rate than types whose offspring have division times $t(d+\delta)$ and $t(d-\delta)$). Nevertheless, results from the simulation model show that asymmetric damage distribution is still favoured (details not shown).

*5. Costs for Asymmetry*

As a further test of model robustness, we assumed that asymmetry itself has costs. To investigate this scenario, we assumed that the survival probability *s(d,r,a)* of a cell with damage *d*, repair *r* and asymmetry a was $s(d)\cdot(1-r/r_{0})\cdot(1-a/a_{0})$ , where *a_0_* is a constant. Costs for asymmetry lead to the emergence of a local fitness maximum without asymmetry (Fig. S1A). However, for intermediate costs of asymmetry, highest fitness, i.e., the global fitness maximum, is still achieved with asymmetry (Fig. S1A). If costs of asymmetry are high enough, the global fitness maximum is achieved with symmetry, while asymmetry is still a local maximum (Fig. S1B).

**Reference (Supplementary Material):**

Charlesworth B (1994). Evolution in Age-Structured Populations. Cambridge: Cambridge University Press.
